# Developing standards of care for HIV prevention research in developing countries – a case study of ten research centers in Eastern and Southern Africa

**DOI:** 10.1186/1742-4690-9-S2-P117

**Published:** 2012-09-13

**Authors:** BP Ngongo, F Priddy, H Park, B Bender, P Fast, O Anzala, G Mutua, E Ruzagira, A Kamali, E Karita, P Mugo, E Chomba, L Bekker, S Roux, A Nanvubya, T Mebrahtu

**Affiliations:** 1 International AIDS Vaccine Initiative, Nairobi, Kenya; 2 Kenya AIDS Vaccine Initiative, Nairobi, Kenya; 3 Medical Research Council – UVRI, Entebbe, Uganda; 4 MRC-UVRI, Entebbe, Uganda; 5 Projet San Francisco, Kigali, Rwanda; 6 KEMRI-CGMRC, Kilifi, Kenya; 7 Zambia Emory HIV Research Program , Lusaka, Zambia; 8 Desmond Tutu HIV Foundation , Cape Town, South Africa; 9 Uganda Virus Research Institute – IAVI, Entebbe, Uganda

## Background

Standards of care in general vary across countries and communities and thus may affect decisions about standards of care provided by research centers in HIV prevention research. To serve as a basis for clarifying and improving standards, a systematic survey of practices at 10 experienced research centers affiliated with the International AIDS Vaccine Initiative in Eastern and Southern Africa was conducted between 2008 and 2010.

## Methods

A survey tool was developed to collect qualitative and quantitative data on types of services provided, recipients of services, referral systems and barriers to referral uptake. Focus group discussion and semi-structured interviews were conducted with key research centre staff. Qualitative data were coded and enumerated where appropriate. Quantitative data were categorized and tabulated using STATA.

## Results

All research centres consistently provided HIV prevention and care services but had varied practices on family planning options, and general medical care to research volunteers, screen out volunteers, partners of volunteers, and former volunteers. Services that were less consistently provided included provision of female condoms and anti-retroviral post-exposure-prophylaxis to volunteers in case of rape. All research centres either had referral points for treatment and care for volunteers who become infected with HIV or provided the services on-site. Limited referral points were available for psychosocial services and adult male circumcision. The greatest challenges for referral uptake included transportation and health care costs, poor quality and inconsistency of services at some referral points. While all research centres covered the cost of health services for study-related adverse events, policies varied on covering the cost of other health services.

## Conclusion

These findings informed the development of standards of care across the 10 research centers and for IAVI-sponsored research. In developing such standards, balance should be made between scientific priorities, considerations of fairness, contextual realities, community expectations, and cost-effectiveness of conducting clinical trials.

